# The Role of Ghrelin and Ghrelin Signaling in Aging

**DOI:** 10.3390/ijms18071511

**Published:** 2017-07-12

**Authors:** Marie Amitani, Haruka Amitani, Kai-Chun Cheng, Timothy Sean Kairupan, Nanami Sameshima, Ippei Shimoshikiryo, Kimiko Mizuma, Natasya Trivena Rokot, Yasuhito Nerome, Tetsuhiro Owaki, Akihiro Asakawa, Akio Inui

**Affiliations:** 1 Education Center for Doctors in Remote Islands and Rural Areas, Kagoshima University Graduate School of Medical and Dental Science, Kagoshima 890-8544, Japan; marisame@m3.kufm.kagoshima-u.ac.jp (M.A.); macchi0625@gmail.com (K.M.); neromekufm@gmail.com (Y.N.); towaki@m2.kufm.kagoshima-u.ac.jp (T.O.); 2 Department of Psychosomatic Internal Medicine, Kagoshima University Graduate School of Medical and Dental Sciences, Kagoshima 890-8544, Japan; satatonma@gmail.com (K.-C.C.); timothykairupan@gmail.com (T.S.K.); samenana@m3.kufm.kagoshima-u.ac.jp (N.S.); natasyarokot@gmail.com (N.T.R.); asakawa@m2.kufm.kagoshima-u.ac.jp (A.A.); inui@m.kufm.kagoshima-u.ac.jp (A.I.); 3 Department of International Island and Community Medicine, Kagoshima University Graduate School of Medical and Dental Sciences, Kagoshima 890-8544, Japan; ippei.35sh@gmail.com

**Keywords:** ghrelin, calorie restriction, longevity, ghrelin resistance, ghrelin agonist

## Abstract

With our aging society, more people hope for a long and healthy life. In recent years, researchers have focused on healthy longevity factors. In particular, calorie restriction delays aging, reduces mortality, and extends life. Ghrelin, which is secreted during fasting, is well known as an orexigenic peptide. Because ghrelin is increased by caloric restriction, ghrelin may play an important role in the mechanism of longevity mediated by calorie restriction. In this review, we will discuss the role of orexigenic peptides with a particular focus on ghrelin. We conclude that the ghrelin-growth hormone secretagogue-R signaling pathway may play an important role in the anti-aging mechanism.

## 1. Introduction

As people age, the risk for aging-related diseases including cancer, cardiovascular disease, and neurodegenerative disease also increases. People desire both a long and healthy life span. The life span of humans has increased in conjunction with the development of modern medicine. The development of a medication to control and suppress aging is in demand [1].

According to Herskind in Denmark, hereditary factors only contribute around 20–30% to a person’s life expectancy [2]. Centenarians, those who live to or beyond the age of 100 years, are thought to have a higher hereditary contribution than non-centenarians [3]. According to a United Nations report in 2015, the number of centenarians in the world has surpassed 450,000 people, four times more than in 1990 [4]. Although the number of centenarians is increasing every year, only a few people have lived longer than 115 years of age. In other words, the average life expectancy of humans has increased, but the maximum life span has not.

During the aging process, most organisms including humans will experience a decrease in physiological and psychological functions. Mechanisms of aging have been proposed, but many details are unknown.

Ghrelin, a 28-amino acid peptide, is the endogenous ligand for growth hormone secretagogue (GHS) and is a main regulator of GH secretion. Ghrelin appears to be involved in several physiological and pathophysiological mechanisms in humans, including aging [5,6]. The known biological activities associated with ghrelin continue to expand. Thus, in this review, we will discuss aging, with a particular focus on its relationship with ghrelin.

## 2. Aging and Longevity

### 2.1. Evolution and Aging

Aging is caused by malfunctions due to irreversible physiological processes, including the accumulation of damage in the living body. Many scientists have used nematodes (*Caenorhabditis elegans* (*C. elegans*)) and yeast (*Saccharomyces cerevisiae*) to explore the aging process. Both of these species have a short life span that leads to easier genetic analysis. In the 1980s, Friedman and Johnson discovered a gene in *C. elegans* called *age-1*, which controls senescence and is related to aging. The study showed that mutations in *age-1* improve the longevity of *C. elegans* [7]. *Age-1* encodes the nematode homolog of phosphatidylinositol 3-kinase (PI3K) and transmits an insulin-like signal [8], which inhibits nuclear transport of the nematode homolog of DAF-16, a member of the forkhead box O (FOXO) family of transcription factors. DAF-16 is activated via a decrease in the insulin-like signal, and modulates a gene cluster that is necessary to control aging [9]. Moreover, scientists have also revealed that both the target of rapamycin (TOR) pathway [10] and the silent information regulator 2 pathway are important for controlling life span [11]. These life span control-signaling pathways are an essential part of longevity control and are also conserved in mammals.

### 2.2. Calories and Longevity

In 1935, McCay first reported the effect of caloric restriction (CR) on life span in rats [12]. CR has been utilized as the most effective experimental method for investigation of the mechanism of aging in geriatrics. Many studies have shown that CR delays most aging-related physiological processes in a variety of species, including mammals and also prevents many aging-related diseases [13]. A long-term study in rhesus monkeys showed that CR extends life span and delays the onset of several pathologic diseases, such as diabetes, cancer, cardiovascular disease, and brain atrophy [14,15]. Many studies have shown that CR decreases oxidative stress, which is thought to be the main mechanism of the aging process [16]. The other mechanisms by which CR controls aging are related to signaling pathways including the Sirtuin (Sir2), insulin-like growth factor 1 (IGF-1), and TOR pathways [17]. Sirtuin is controlled by nicotinamide adenine dinucleotide (NAD), which mediates metabolism. The insulin-like signal is controlled by glucose, and the TOR signal is controlled by amino acids and ATP. In young rats, CR decreases GH secretion and the plasma GH concentration [18].

A clinical study of persons 100 years old or older in Okinawa suggested that CR leads to longevity and well-being [19]. Since the first evidence that CR extends life span and suppresses age-related chronic diseases was presented, numerous studies have also reported the relationship between body weight and mortality. Being overweight is associated with an increased risk of total mortality compared with being of normal weight [20].

Diet-induced obesity causes ghrelin resistance, which is improved by weight loss due to CR [21]. After 24 months, mood also clearly improved in the group with CR compared with a free feeding group. CR reduces tension and improves general health and sex drive [22]. Ghrelin resistance also occurs in elderly persons [23].

### 2.3. IGF-1 and Other Age-Related Factors

#### 2.3.1. GH and IGF-1

Human aging is related to a change in GH/IGF-1 activity. The IGF-1 receptor is encoded by *daf-2* [24]. *Age-1* and a *daf-2* variant in the nematode result in a life span that is 2–3 times longer than that of the wild type. Age-1 transmits an insulin-like signal [8]. *Daf-2* has homology with an insulin receptor gene in the human genome and the IGF-1 receptor gene. [25]. A similar result was seen in yeast and *Drosophila*, in which life span is extended in a genetic variant with functional deletion of a component of the insulin/IGF-1-like signaling pathway [26,27]. In addition, CR also reduces the plasma IGF-1 concentration [28].

Circulating levels of GH, IGF-1, and ghrelin decline with aging, and aging may impair endogenous ghrelin signaling. Aging is characterized by a decrease in somatotroph cell functionality involving GH-releasing hormone receptor. Many studies have suggested that GH is a key factor in aging. The age-related decline in the activity of the GH/IGF-I axis is considered to contribute to age-related changes.

The efficacy of GH and/or ghrelin therapy in animal models and clinical studies has been reported. Administration of human GH for six months was accompanied by an 8.8% increase in lean body mass, a 14.4% decrease in adipose-tissue mass, and a 1.6% increase in average lumbar vertebral bone density [29]. Another study indicated the effect of combined therapy with ghrelin and GH for repair of organ injury and survival and improved immune function in septic aged animals [30]. GH levels increase when a physically unimpaired person is given the ghrelin agonist, anamorelin 25 mg orally, although the increase is minimal in young males and females compared with elderly males and females [23].

Although anti-aging effects of GH have been reported, the risk of the use of GH in healthy persons is still unknown. Whether or not GH deficiency constitutes a beneficial adaptation to aging or can serve as an anti-aging therapy is unclear [29,31], despite an article showing that continued GH therapy leads to unacceptable side effects. GH therapy in healthy elderly persons has not been thoroughly explored, but data suggest that GH treatment is associated with small changes in body composition and increased rates of adverse events. Thus, GH cannot be recommended as an anti-aging therapy [32]. In addition, in 2009, the Growth Hormone Research Society presented conclusions regarding the use of GH and GHS for promoting life span. They stated that until future carefully designed, long-term clinical studies with validated outcome parameters have been conducted, the clinical use of GH or GHS in older adults, alone or in combination with testosterone, cannot be recommended [33]. 

#### 2.3.2. Sirtuin

*Sir2*, a NAD-dependent histone deacetylase, controls the life of the mother cell of the yeast fungus. In experimental models, a variant with inactivated *Sir2* is short-lived, and a variant that overexpresses *Sir2* has a longer life span than the wild type [34]. *Sir2* also controls the life span in individual nematodes and *Drosophila* [35,36]. In Sir2 knockout models of yeast fungus, nematodes, and *Drosophila*, the life extension effect of CR is inhibited [36,37]. The Sir2 homolog in mammals is called *Sirtuin 1* (*Sirt1*). *Sirt1* is associated with various effects of CR, including an increase in life span. Sirt1 may affect neuropeptide Y (NPY)/agouti-related protein (AgRP)-positive neurons and metabolism. Sirt1 deacetylates other important proteins such as histones, p53, NF-κB, FOXO, and peroxisome proliferator-activated receptor-gamma coactivator 1 alpha (PGC-1α) [38]. In transgenic mice that overexpress *Sirt1*, glucose tolerance is high, and a healthy metabolic state is present, similar to what is seen in calorie-restricted mice [39]. The hypothalamic melanocortin system is affected by *Sirt1*, which promotes the activity and connectivity of this system, resulting in a negative energy balance. On the other hand, in Sirt1 knockout mice, life extension due to CR is not seen [40]. Sirt1 is necessary to control energy balance related to aging and longevity, and plays an important role in the normal response to CR [41,42,43]. In an animal model, inhibition of brain Sirt1 activity decreases AgRP neuronal activity and inhibits proopiomelanocortin (POMC)-positive neurons. A Sirt1 inhibitor decreases food intake via melanocortin receptors, and the melanocortin 4 receptor (MC4R) antagonist reverses the effect of Sirt1 on food intake. This activity of Sirt1 is related to the mitochondrial protein, UCP2. In addition, knockout of *Sirt1* in hypothalamic AgRP neurons decreases the electric responses of AgRP neurons to ghrelin and decreases food intake, leading to decreased lean mass, fat mass, and body weight [44].

#### 2.3.3. Klotho

Klotho is expressed mainly in the kidney, parathyroid gland, and brain [45]. Overexpression of Klotho extends life span, and inhibition of Klotho promotes aging. Klotho-deficient mice (kl^−/−^) present with symptoms similar to aging, including growth retardation, hypogonadotropic hypogonadism, skin atrophy, sarcopenia, vascular calcification, osteopenia, cognitive impairment, pulmonary emphysema, and death at approximately two months of age [46,47,48]. Klotho reduces oxidative stress through phosphate metabolism [47]. In addition, Klotho plays an important role in the bone-kidney endocrine axis and acts on fibroblast growth factor-23 [49]. The increased mortality in overweight individuals may be correlated with the level of Klotho gene expression in these people [50].

#### 2.3.4. TOR

TOR is a serine-threonine kinase that is highly expressed in yeast, plants, nematodes, flies, mice, and humans [51]. TOR is the catalytic subunit of two protein complexes, TORC1 (TOR complex 1) and TORC2 (TOR complex 2). The differences between these two complexes are in their protein structures, rapamycin sensitivity, activated signal of upper reach, and downstream output. TOR unifies various intracellular environmental factors such as insulin and other growth factors, the energy state, and the oxidation-reduction state. TOR also controls growth, survival, and proliferation of the cell through transcription and translation [52,53,54]. In nematodes, knockout of TOR and Raptor (TORC1 subunit) leads to life span extension [10,55]. Life span is thought to be extended by mimicking the effect of CR. Life span is also extended by knockdown of S6 kinase, which is one of the targets of TOR [56]. CR increases the number of intestinal stem cells through mTOR1 (mammalian TOR 1) and Sirt1. Thus, rapamycin, which is an mTOR1 inhibitor, may block the action of CR in intestinal stem cells [57].

## 3. Ghrelin and Aging

### 3.1. Ghrelin and NPY

The hypothalamus plays an important role in aging. In the hypothalamus, complex neural networks regulate homeostasis including appetite control. Many factors are implicated in the hypothalamic regulation of food intake, including melanin-concentrating hormone, NPY, AgRP, POMC, and cocaine- and amphetamine-regulated transcript. Ghrelin is a peripheral hormone with orexigenic properties and is part of a regulatory feedback loop between the periphery and brain. Among the numerous circulating appetite-regulating peptides, these two orexigenic peptides, NPY and ghrelin, are important factors in aging [58].

One hypothesis states that aging is associated with attenuated ghrelin signaling. CR produces health benefits accompanied by enhanced ghrelin production. Ghrelin is present in two forms: unacylated ghrelin; des-acyl ghrelin and acylated ghrelin; acyl ghrelin. CR increases plasma acyl ghrelin and des-acyl ghrelin concentrations in an animal model [59]. In addition, CR increases expression of hypothalamic AgRP and NPY, in contrast to reduced expression of POMC [60].

Recently, scientists have focused on the effect of NPY and ghrelin in autophagy in cortical neurons and their involvement in CR-induced autophagy. CR stimulates autophagy and increases NPY and ghrelin at the same time. NPY, an orexigenic peptide, decreases with aging. One of the causes of aging is impaired autophagy. CR mimetic cell culture medium stimulates autophagy. Ghrelin promotes autophagy via NPY in the hypothalamus, and exogenous NPY or ghrelin stimulates autophagy. On the other hand, NPY or ghrelin receptor antagonists block this effect. Impaired autophagy occurs in aging and age-related neurodegenerative diseases, and NPY and ghrelin mediate the neuroprotective effects induced by CR [61].

Long-term CR also mediates NPY receptor subtype density in rats. NPY and ghrelin may participate in autophagy adjustment through NPY Y1 and Y5 receptors in the hypothalamus [61,62,63,64]. The life span of mice is extended when they are fed with 70% of free food intake. However, the life span is not extended in similarly fed NPY knockout mice [65]. Oxidative stress tolerance and the tumor suppressant effect of CR are also attenuated in NPY knockout mice.

Ghrelin is secreted from the stomach during fasting [66] and has various physiologic functions in addition to its role as an orexigenic hormone. In the hypothalamus, ghrelin promotes expression of AgRP and NPY. Ghrelin stimulates GH secretion [67], gastrointestinal movement [68], and heart systole [69]. Ghrelin also controls energy metabolism [66], insulin secretion [70,71], inflammation [72], apoptosis, cardiovascular function, immune function, and neurodegeneration [73]. Ghrelin stimulates secretion of GH through growth hormone secretagogue receptor (GHS-R). Two isoforms, GHS-R1a and GHS-R1b, have been identified. GHS-R1a binds acyl ghrelin and transduces its message to induce GH secretion and stimulate secretion of IGF-1 from the liver. GHS-R1a is expressed widely in the pituitary, hypothalamus, pancreas, adipose tissue, immune cells, and the cardiac system. Thus, GHS-R1a agonists or antagonists may serve as novel and effective therapeutic options for many syndromes and diseases, such as cancer cachexia, aging-related cognitive decline, obesity, and diabetes [74].

The orexigenic effect of ghrelin due to CR is caused by activation of adenosine monophosphate-activated protein kinase (AMPK) in NPY neurons in the hypothalamus arcuate nucleus. Ghrelin activates AMPK, which increases NAD^+^ and stimulates Sirt1 activity. Ghrelin secretion due to CR is neuroprotective of dopaminergic neurons [75].

Several studies have suggested that age and obesity decrease the circulating acyl ghrelin levels [75,76,77]. Ablation of ghrelin signaling inhibits liver steatosis, which is related to reduce peroxisome proliferator-activated receptor (Ppar)-γ expression and enhanced insulin receptor substrate 2 (Irs2) expression. This study indicated that the effect of CR depends on enhanced metabolic flexibility independent of endogenous ghrelin or des-acyl ghrelin signaling [76].

In the absence of melanocortin-3 receptors, lower AgRP/NPY expression, attenuated food anticipatory activity could enhance circuitry regulating anticipatory responses to nutrient loading were seen, which suggest the importance of melanocortin-3 receptors as modulators of anticipatory responses to feeding [77]. Ghrelin signaling is an important thermogenic regulator in aging. During aging, plasma ghrelin and GHS-R expression in brown adipose tissue are increased. Increased plasma ghrelin during aging may lead to an imbalance in thermogenic regulation, which may in turn exacerbate impaired thermogenic regulation in aging [78]. AgRP neurons are key sites for GHS-R-mediated thermogenesis, and GHS-R in AgRP neurons play crucial roles in governing energy utilization and pathogenesis of diet-induced obesity [79].

Ghrelin might be a target for potential anti-obesity therapies. In obese patients, ghrelin levels were negatively associated with fasting insulin and HOMA-IR [80]. Leptin has been implicated as the antagonist in leptin and ghrelin systems. Leptin induces inhibition of AgRP and NPY expression, which is opposite to the effect of ghrelin [81]. Recessive mutations of the *ob* gene lead to accelerate morbid obesity and metabolic disorders, resulting in early mortality and a shortened life span. Obese *ob/ob* mice with enhanced leptin transgenic expression show a life span that is more than double that of control obese *ob/ob* mice, which have a life span similar to that of normal wild-type mice [82]. However, *ob/ob* mice fed a high fat diet (HFD) remain sensitive to ghrelin, which indicated that hyperleptinaemia, instead of obesity or a HFD, causes ghrelin resistance [83]. The life-extending benefits of leptin are associated with drastic reductions in visceral fat, blood glucose, and insulin levels, but elevated ghrelin levels. Thus, leptin derived from ectopic gene expression in the hypothalamus alone is both necessary and sufficient to normalize the life span.

An investigation of age-related metabolic changes showed that plasma acyl ghrelin levels are lower in young mice, whereas leptin levels under normal feeding conditions are substantially higher in old mice. The expression levels of hypothalamic preproghrelin under normal feeding conditions and the expression levels of NPY and AgRP under fasting conditions are lower compared with those of young mice [84].

Another study has investigated clinical biomarkers that distinguish between long-lived and short-lived individuals [85]. Compared with “long-lived” participants (older than 90 years), no significant single biomarker, including ghrelin, insulin, leptin, interleukin-6, adiponectin, or testosterone, was found in “short-lived” participants (72–76 years of age) [85]. Ghrelin or leptin may not be an effective single biomarker of health, and a combination of multiple biomarkers is likely needed.

Recently, the physiological function of butyrylcholinesterase (BChE) as a ghrelin hydrolase has been reported [86]. BChE converts acyl ghrelin into des-acyl ghrelin. High levels of BChE predict long-term survival of patients with coronary artery disease [87]. BChE regulates ghrelin and affects emotional behavior and life span in relation to ghrelin [88]. Overexpression of BchE induces low ghrelin levels and reduced aggression and social stress in mice [88]. One hypothesis is that elderly and obese individuals have ghrelin resistance, which is a key factor in aging. However, the efficacy of ghrelin for promoting longevity remains controversial. Further clinical studies are needed to clarify the mechanism of longevity.

### 3.2. Sarcopenia and Frailty

Ghrelin and ghrelin receptor agonists also improve skeletal muscle atrophy. Ghrelin shows anti-catabolic and anti-inflammatory effects, and leads to inhibition of catabolism of muscle proteins [89]. Age-related changes in muscle influence longevity and a healthy life expectancy. Ghrelin is effective in preventing sarcopenia and muscle atrophy in cancer cachexia [90]. Thus, ghrelin has been proposed as a treatment for sarcopenia. Ghrelin also prevents tumor implantation and cisplatin-induced muscle atrophy in vivo and in vitro, significantly increasing muscle mass and grip strength and improving survival. Ghrelin prevents muscle atrophy by down-regulating inflammation and p38/C/EBP-β/myostatin, and activating Akt, myogenin, and myoD [91].

Recently, an effect of des-acyl ghrelin on muscle has been reported. Des-acyl ghrelin induces skeletal muscle regeneration after ischemia via superoxide dismutase-2-induced miR-221/222 expression [92]. Des-acyl ghrelin restores the impaired insulin and autophagic signaling in the skeletal muscle of diabetic mice [93]. These studies indicate a preventive and repair effect of des-acyl ghrelin on skeletal muscle damage [93].

Aging is commonly associated with low-grade adipose inflammation and insulin resistance. Frailty is associated with an altered glucose-insulin axis. Ghrelin signaling plays an important role in macrophage polarization and adipose tissue inflammation during aging. Expression of GHS-R increases in adipose tissues during aging, and old Ghsr ^(−/−)^ mice exhibit a lean and insulin-sensitive phenotype [94]. It indicated to consider the use of ghrelin signaling antagonist to improve the body’s metabolism. However, recently study showed that ghrelin deficiency does not affect longevity in mice [95].

Elderly individuals with sarcopenia show significantly lower ghrelin levels than those without sarcopenia, but these differences disappeared when individuals were stratified by gender. The ghrelin levels of elderly subjects without sarcopenia are not decreased compared with young adults [96]. In addition, administration of an oral ghrelin mimetic to healthy older adults increases the total body weight and lean body mass, although no significant difference in muscle strength or quality of life was found [97]. In another study, administration of an oral ghrelin agonist increased tandem walking and stair climbing, as well as lean body mass [98]. Frail women have higher fasting levels of free fatty acid (FFA), resistin, GH, and interleukin-6 and lower fasting levels of ghrelin, adiponectin, glucagon-like peptide 1 (GLP-1), and IGF-1 compared with non-frail women, although the differences were not statistically significant [99].

The mutual interplay among energy homeostasis, acyl ghrelin and des-acyl ghrelin, and bone metabolism is also an important concept. Regulatory networks may exist between the orexigenic ghrelin pathway and bone metabolism, which is age dependent. Increases in food intake and direct effects on muscle proteolysis and protein synthesis are likely to mediate these effects, but the pathways leading to these events are not well understood. Acyl ghrelin inhibits osteoclast formation and induces osteoprotegerin gene expression [100]. Ghrelin and leptin antagonize each other and metabolically balance each other. Leptin suppresses osteoclastogenic activity via ghrelin receptors in a leptin-deficient animal model [101]. Additionally, osteoporosis in humans using growth hormone secretagogue receptor (GHSR) agonists has had minor success in post-menopausal women [102]. These studies suggest that the metabolic pathway, ghrelin, and leptin play an important role in frailty syndrome and bone metabolism.

### 3.3. Ghrelin and Memory

Increasing evidence suggests an association between ghrelin and Alzheimer’s disease pathology. Ghrelin therapy may be a potential strategy for preventing or treating neurodegenerative diseases. Previous studies have shown that ghrelin also affects mood, anxiety, cognition, and memory retention in addition to its role in metabolism and energy intake [103]. The hippocampus, amygdala, and dorsal raphe nucleus play a role in cognition [104,105], and the central serotonin system plays an important role in anxiety and memory. Memory retention is induced by ghrelin, whereas GHS-R1 depletion decreases serotonin activity [106,107,108]. In addition, decreased memory is improved by acute ghrelin administration [109,110]. Acute central administration of ghrelin to mice increases serotonergic turnover in the amygdala by affecting mRNA expression of a number of serotonin receptors, both in the amygdala and in the dorsal raphe [111]. Moreover, ghrelin mediated circadian rhythms via food intake in a recent study [112]. Ghrelin drives higher-order feeding processes related to food reward, food seeking, and learned and motivational aspects of feeding via CNS signaling through GHS-R1a [113]. A recent study suggested that ghrelin accelerates neurogenesis and activity development in cultured cortical networks [114]. Ghrelin increases survival and reduces cell death of hippocampal neurons and shows neuroprotective effects [115].

Ghrelin accelerates hippocampal synaptic plasticity and increases spatial memory via activation of PI3K [116]. Ghrelin administration affects long-term spine density of neurons in the hippocampus [117]. Acyl ghrelin expression in the amygdala is related to spatial learning through GHS-R1 [118]. GHS-R1a knockout mice exhibit improvements in spatial memory and deficits in contextual memory [119]. GHS-R1a is required for contextual memory, and GHS-R1a affects acquisition of spatial memory in the open field test and Morris water maze [119].

The neuroprotective mechanism of ghrelin in aging is considered to be an increase in Sirt1 activity in the brain and a decrease in microglial activity. An experiment to investigate an association between ghrelin and a ghrelin agonist and longevity was conducted using Klotho-deficient mice, senescence-accelerated mouse prone 8 (SAMP8 mice), and ICR mice. These three types of mice are models of accelerated senescence and aging. SAMP8 mice show an age-related increase in β-amyloid and a similar phenome as Alzheimer’s disease model mice. The ghrelin receptor antagonist (D-Lys3)-GHRP-6 hastens death. Furthermore, the ghrelin agonists, rikkunshito and atractylodin, activate Sirt1, improve aging-related disease, and extend life span [58].

## 4. Effect of CR Mimetics and Ghrelin Agonists on Longevity

Many studies have been conducted on compounds with similar actions as CR including activation of Sirt1 and extension of life span (Table 1). Based on preliminary study results, the use of ghrelin mimetics may be more suitable for use in elderly individuals than GH itself. Resveratrol, a polyphenol found in grapes, is a representative CR mimetic. Rapamycin, 2-deoxy-d-glucose, and metformin also show similar effects as CR. The life span of mice is shortened by intake of a high-fat diet. However, when resveratrol, which activates Sirt1, was given, the life span of the high-fat diet-fed mice was not shortened [120]. The life span is not extended even if resveratrol is given to mice fed a normal diet. Mice given resveratrol show a gene expression pattern like that induced by CR, including maintenance of elasticity of blood vessels, and anti-aging effects such as a delay in the onset of cataracts and increased exercise ability [121]. Recently, the amino acid sequence of Sirt1 has been determined, and its active mechanism has been elucidated [122].

Rapamycin, which is an immunosuppressive drug, extends the life span of nematodes and yeast via TORC1 inhibition. Rapamycin also extends the life span of mice by inhibiting mTOR1, and this drug reduces phosphorylation of ribosomal protein S6 kinase 1 (S6K1), which is a main downstream effecter of mTOR1 [123]. In addition, rapamycin extends the life span of S6K1 knockout mice [124].

Rikkunshito, a traditional Japanese kampo, is composed of 43 ingredients. Rikkunshito is used in Japan to treat upper gastrointestinal symptoms in patients with functional dyspepsia (gastroesophageal reflux disease), dyspeptic symptoms in postgastrointestinal surgery patients, chemotherapy-induced dyspepsia in cancer patients, cancer cachexia syndrome, and anorexia due to aging. Oral administration of rikkunshito potentiates the orexigenic action of ghrelin through several different mechanisms [125]. In this review, we will highlight what is known about the orexigenic effect of rikkunshito with a special focus on the interaction with the ghrelin signaling system [126].

Dysregulation of ghrelin secretion and ghrelin resistance in the appetite control system occurs in aged mice. Rikkunshito ameliorates aging-associated anorexia via inhibition of phosphodiesterase 3 (PDE3) [84]. The effects of ghrelin, rikkunshito, PDE3, and PDE3 kinase inhibitors on appetite have been studied. Although ghrelin supplementation (33 μg/kg) failed to increase food intake in aged mice, oral administration of a PDE3 kinase inhibitor and a PDE3 inhibitor increases food intake in aged mice [84]. Rikkunshito also increases food intake in aged mice. Rikkunshito stimulates ghrelin secretion via serotonin2b/2c receptor antagonism, inhibits the ghrelin metabolic enzyme, improves ghrelin resistance [127], and reinforces ghrelin signaling by increasing GHS-R activity [128]. Rikkunshito promotes ghrelin secretion and reinforces ghrelin sensitivity in both the central and peripheral nervous systems.

Certain components of rikkunshito (nobiletin, isoliquiritigenin, and heptamethoxyflavone) have inhibitory effects on PDE3 [84]. Atractylodin is another main ingredient of rikkunshito, and it is detected as a major component in the plasma of volunteers given rikkunshito. Atractylodin extends the life span of Klotho-deficient mice. Atractylodin increases the calcium concentration in cells when ghrelin is added to cells that express GHS-R. Thus, rikkunshito and atractylodin show similar actions as CR via ghrelin-GHS-R signaling.

Rikkunshito enhances phosphorylation of cyclic adenosine monophosphate (cAMP) response element binding protein through the ghrelin receptor and activates Sirt1. Interestingly, an active increase in Sirt1 was not seen if rikkunshito was given to ghrelin receptor knockout mice, suggesting that rikkunshito activates Sirt1 through ghrelin and the ghrelin receptor, leading to an extended life span. Rikkunshito increases Sirt1 activity through the ghrelin receptor and significantly extends the life span in three models of accelerated senescence and aging: Klotho-deficient mice, SAMP8 mice, and ICR mice [58].

## 5. Conclusions

**Figure 1:**
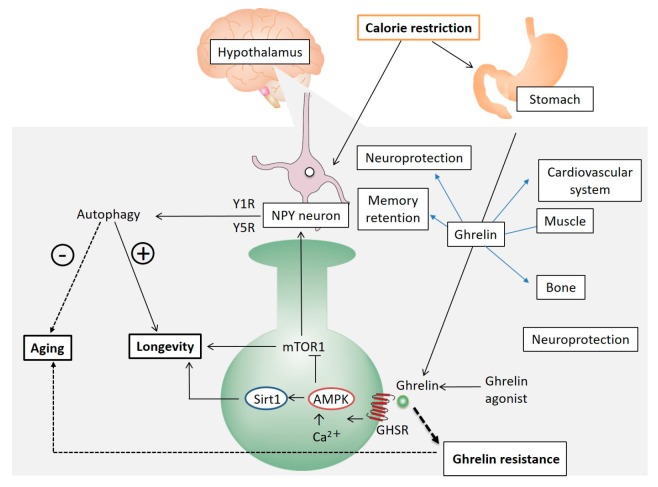
CR increases the expression of hypothalamic AgRP and NPY and reduces the expression of POMC. NPY promotes autophagy in the hypothalamus. Ghrelin also affects the cardiovascular system, muscle, bone, and memory retention, as well as providing a neuroprotective effect, all of which result in an extended life span and anti-aging effects. CR: caloric restriction; AgRP: agouti-related protein; POMC: proopiomelanocortin; NPY: neuropeptide Y.

We discussed the role of orexigenic peptides with a particular focus on ghrelin. Ghrelin-GHS-R signaling may play an important role in the mechanism of aging (Figure 1). CR mimetics and ghrelin mimetics may provide new hope for improving a healthy life expectancy in our aging society. However, controversy remains regarding the efficacy of ghrelin for enhancing longevity. Further clinical studies are needed to clarify the mechanism of longevity.

## Figures and Tables

**Table 1:** Ghrelin and longevity.

Formula	Model	Reported Outcome	Study	References
Ghrelin	Rat cortical neurons	Stimulation of network formation and activation in cortical neuronal networks	In vitro	Veyrat-Durebex C et al., 2013 [64]
Ghrelin and Ghrelin antagonist	Rat cortical neurons	Caloric restriction mimetic cell culture medium stimulated autophagy in rat cortical neurons and ghrelin receptor antagonists blocked this effect. On the other hand, exogenous ghrelin stimulated autophagy in rat cortical neurons.	In vitro	Ferreira-Marques M et al., 2016 [61]
Ghrelin	Normal rats	Increase in memory retention	In vivo	Carlini VP et al., 2002 [103]
Ghrelin	SAMP8 (Alzheimer’s disease model)	Improvement of retention on the T-maze foot shock avoidance task	In vivo	Diano et al., 2006 [108]
Ghrelin	Cerebral ischemia/reperfusion rat model	Increase in survival and reduce cell death of hippocampal neurons following ischemia/reperfusion injury	In vivo	Liu Y et al., 2006 [115]
Ghrelin	Normal rats	SSRI decreased the effects of ghrelin on memory retention	In vivo	Carlini VP et al., 2007 [106]
Ghrelin	Normal mice	Increase in the impaired memory of mice with 50% food restriction	In vivo	Carlini VP et al., 2008 [110]
Ghrelin mimetic	6- and 75-week-old C57BL/6J mice	Amelioration of aging-associated anorexia in mice via inhibition of PDE3	In vivo	Takeda et al., 2010 [84]
Ghrelin agonist and mimetic	Klotho-deficient, SAMP8 and ICR mice	Decrease in microglial activation in the brain and prolongation of survival in klotho-deficient, SAMP8 and aged ICR mice	In vitro and vivo	Fujitsuka et al., 2016 [58]
Ghrelin and GH	Septic aged rats	Prevention of the loss of splenic T cells and improvement of sepsis-induced immunosuppression	In vivo	Zhou et al., 2017 [30]
Ghrelin	Obese women	Obesity-linked reductions in ghrelin were reversed by weight loss achieved through caloric restriction	Clinical	Bayliss JA et al., 2016 [75]
Ghrelin mimetic	Healthy older adults, randomized, double-blind, placebo-controlled study	Increase in total body weight and lean body mass. However, no significant difference in muscle strength, function and quality of life	Clinical	Nass R et al., 2008 [97]
Ghrelin agonist	Healthy older adults, randomized, double-blind, placebo-controlled study	Increase in lean mass, tandem walk and stair climb	Clinical	White HK et al., 2009 [98]
BChE	Patients with coronary artery disease	Presentation of CAD affected the effect of BChE on mortality	Clinical	Goliasch G et al., 2012 [87]
Ghrelin	Healthy older adults, cohort study	Ghrelin measured during an OGTT predicted major health events and death in older adults	Clinical	Kaplan RC et al., 2017 [6]

SAMP8, senescence-accelerated mouse prone/8; SSRI, selective Serotonin Reuptake Inhibitors; PDE3, phosphodiesterase 3; ICR, intermittent calorie restriction; GH, Growth hormone; CAD: coronary artery disease; BChE, butyrylcholinesterase; OGTT: oral glucose tolerance test.

## Abbreviations

AgRP	Agouti-related protein
AMPK	Adenosine monophosphate-activated protein kinase
cAMP	Cyclic adenosine monophosphate
CR	Calorie restriction
FFA	Free fatty acid
FOXO	Forkhead box O
GHSR	Growth hormone secretagogue receptor
GHSR-1a	Growth hormone secretagogue receptor-1a
GLP-1	Glucagon-like peptide 1
HFD	High fat diet
IGF-1	Insulin-like growth factor 1
Irs2	Insulin receptor substrate 2
NAD	Nicotinamide adenine dinucleotide
NPY	Neuropeptide Y
MC4R	Melanocortin 4 receptor
mTOR1	Mammalian TOR 1
PI3K	Phosphatidylinositol 3-kinase
PGC	Peroxisome proliferator-activated receptor-gamma coactivator
POMC	Proopiomelanocortin
Ppar	Peroxisome proliferator-activated receptor
SAMP8	Senescence-accelerated mouse prone/8
S6K1	Ribosomal protein S6 kinase 1
TOR	Target of rapamycin
TORC1	TOR complex 1
TORC2	TOR complex 2
